# Design of SARS-CoV-2 Main Protease Inhibitors Using Artificial Intelligence and Molecular Dynamic Simulations

**DOI:** 10.3390/molecules27134020

**Published:** 2022-06-22

**Authors:** Lars Elend, Luise Jacobsen, Tim Cofala, Jonas Prellberg, Thomas Teusch, Oliver Kramer, Ilia A. Solov’yov

**Affiliations:** 1 Computational Intelligence Lab, Department of Computer Science, Carl von Ossietzky University, Ammerländer Heerstraße 114-118, 26129 Oldenburg, Germany; lars.elend@uni-oldenburg.de (L.E.); tim.cofala@uni-oldenburg.de (T.C.); jonas.prellberg@uni-oldenburg.de (J.P.); 2 Department of Physics, Chemistry and Pharmacy, University of Southern Denmark, Campusvej 55, 5230 Odense M, Denmark; luja@sdu.dk; 3 Department of Physics, Carl von Ossietzky University, Carl-von-Ossietzky-Str. 9-11, 26129 Oldenburg, Germany; thomas.teusch@uni-oldenburg.de; 4 Research Center for Neurosensory Science, Carl von Ossietzky Universität Oldenburg, 26111 Oldenburg, Germany; 5 Center for Nanoscale Dynamics (CENAD), Carl von Ossietzky Universität Oldenburg, Institut für Physik, Ammerländer Heerstr. 114-118, 26129 Oldenburg, Germany

**Keywords:** drug design, artificial intelligence, neural networks, evolutionary algorithms, molecular dynamics, SARS-CoV-2

## Abstract

Drug design is a time-consuming and cumbersome process due to the vast search space of drug-like molecules and the difficulty of investigating atomic and electronic interactions. The present paper proposes a computational drug design workflow that combines artificial intelligence (AI) methods, i.e., an evolutionary algorithm and artificial neural network model, and molecular dynamics (MD) simulations to design and evaluate potential drug candidates. For the purpose of illustration, the proposed workflow was applied to design drug candidates against the main protease of severe acute respiratory syndrome coronavirus 2. From the ∼140,000 molecules designed using AI methods, MD analysis identified two molecules as potential drug candidates.

## 1. Introduction

Drug discovery is an important field of study that ensures the ability to continuously combat emerging diseases. The goal in drug discovery is to identify molecules (ligands) with the ability to bind to a macro-molecule (receptor) and consequently block the expression or development of a targeted disease [1,2,3]. While experimental drug discovery provides important information on potential drugs, atomic-level details are inaccessible to experimental studies [4]. Computer-aided drug design (CADD) tools, e.g., molecular docking, quantum chemical methods, and molecular dynamics (MD) simulations can be applied to obtain information about the interactions taking place on the atomic- and electronic-level that governs the binding affinity between a ligand and a receptor, e.g., electrostatic and van der Waals (vdW) interactions, as well as the conformational changes of both the ligand and the receptor due to their interaction.

The search for a suitable drug candidates is a complicated process. While designing a potent drug, the medicinal chemists face a complex multidimensional optimization problem, balancing between various desired molecular properties, such as the biological activity, absorption, toxicity and the availability of the compound [5]. The search space of possible drug molecules is enormous (there are at least $10^{60}$ molecules with less than 500 g/mol in the universe) [6], and hence a complete exploration of the search space of all potent drug molecules is infeasible. Incorporating methods from the domain of artificial intelligence (AI) into drug discovery processes can help systematically traverse this search space. Training on databases of already known drugs allows identifying patterns in the nature of these molecules and generating new molecules with similar properties. Furthermore, evolutionary approaches can be utilized to optimize existing molecules with respect to desired metrics.

There are numerous examples in the literature of how AI is being used to assist in the drug development process, a short overview of which is provided in the following. A taxonomy of *de novo* drug design methods are given by Vasundhara et al. [7] and Brown et al. [8]. Some approaches concentrate on the design of molecules from atoms [9,10], while others use chemical fragments as their smallest building blocks [11]. Further work [11,12] aims to find drugs that bind to a specific protein binding site. The approach presented in the current study is based on an evolutionary algorithm (EA), a nature-inspired optimization strategy, adapted for drug design [13]. The EA is augmented with a neural language model, which is trained on a database of drug-like molecules, to improve the quality of the generated drug candidates.

While CADD and AI methods can be applied in any arbitrary ligand-receptor complex, a currently highly relevant example is the main protease (M^pro^), also known as the 3C-like protease (3CL^pro^), of severe acute respiratory syndrome coronavirus-2 (SARS-CoV-2), which is responsible for the coronavirus disease 2019 (COVID-19) pandemic. M^pro^ is responsible for the cleavage of the viral polyprotein and is, therefore, vital for the SARS-CoV-2 life cycle [14]. Because M^pro^ simultaneously has a low resemblance to related human homologies, it is a potent drug target for the treatment of coronaviruses [14,15,16]. Specifically, it is of interest to find a drug that inhibits the M^pro^ cleavage site and thereby blocks the viral replication.

CADD approaches are typically divided into two main categories, which consider (i) existing known molecules from large databases, such as the ZINC database [17] or the DrugBank database [18], as the possible drug candidates; (ii) new molecules that are engineered, based on existing molecular datasets. Liu et al. [19] provides a comprehensive review of CADD for SARS-CoV-2 protein inhibitor candidate discovery, with numerous examples for both approaches. Many papers use existing molecules and investigate their usefulness as SARS-CoV-2 inhibitors, using docking and MD simulations [20,21,22]. Arshia et al. [23] also discussed an approach combining AI with subsequent MD simulations for the design of SARS-CoV-2 protease inhibitor candidates. In that study, the AI methodology employed LSTM neural network for generating novel potential drug molecules and mainly considered the binding affinity as the optimization metric.

**Figure 1:**
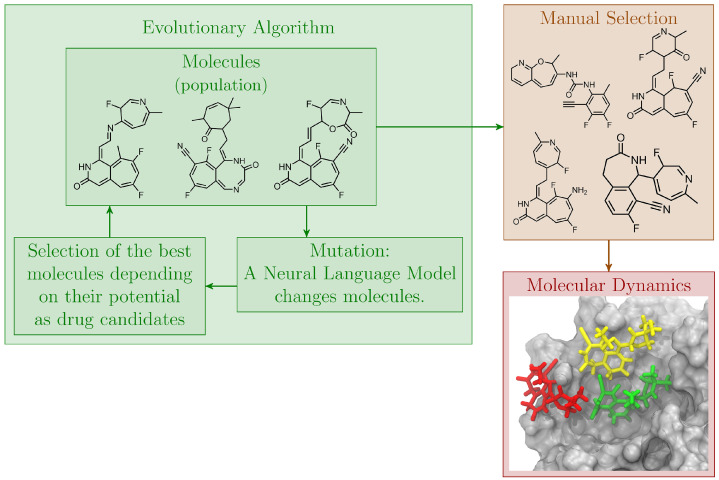
Overview of the proposed drug design workflow. An Evolutionary Algorithm (EA) generates drug candidates by iteratively mutating populations of molecules using a language model. The drug candidacy of the molecules is evaluated using fitness metrics. The best molecules, determined by the EA and a subsequent manual selection, are characterized further by molecular dynamics simulations.

The current paper introduces a computational drug design workflow that combines AI governed drug design and the CADD methods of molecular docking and atomistic MD simulations. The workflow is first introduced in detail, and is further illustrated through a study of drug molecules interacting with M^pro^ of SARS-CoV-2. M^pro^ is a promising drug target because it is conserved across different variants within the Coronaviridae [24]. This makes M^pro^ an interesting drug target also for mutations of the virus, since any change in the function of this protein could be fatal for the virus [25]. We use M^pro^ as the main example to demonstrate the proposed approach. It should however be noted that the approach could also be applied to other targets and viruses in the future. Since the main focus of this paper is on introducing the novel AI-MD approach, the detailed discussion of potent drugs against SARS-CoV-2 and its mutants is left open for further studies. Figure 1 shows a schematic overview of the proposed workflow, which initially applies AI-based methods to design a list of potential drug candidates targeting M^pro^. The potency of the designed molecules is evaluated based on preliminary binding affinities computed by QuickVina 2 [26] and heuristic drug design metrics. Subsequently, MD simulations are performed of the most potent ligands binding to M^pro^ to gather more detailed information on the ligand-receptor interactions and the dynamical behavior of the complex. The AI part generates efficiently many thousands of molecules that can act as potential inhibitors, while the MD part provides a simple way to validate and thus narrow down these potential inhibitors before they can be further investigated in a wet lab in the future. The proposed workflow is discussed in detail below.

## 2. Methods

This section presents the general concept of the proposed AI-MD workflow and provides the necessary details for the considered case study, that was used to benchmark the method. First, the molecule design metrics are introduced, that are then used in the evolutionary molecule generation algorithm. Finally, the concept of MD is presented, and it is explained how it should be coupled with the AI approach.

### 2.1. Molecule Design Metrics

Potential drug candidates were optimized with respect to metrics that estimate how likely a drug candidate is to act as an inhibitor (for example in the case of M^pro^). The metrics considered in the optimization process were motivated by a previous study [13]. The score ranges and the optima of the metrics are shown in Table 1 and are further described below.

#### 2.1.1. Binding Affinity (BA)

The BA score estimates the binding free energy between the receptor and a potential ligand. The BA score was computed using the AutoDock Vina (Vina) [27] based QuickVina 2 [26] docking software that uses a hybrid scoring function based on empirical and knowledge-based data [27]. Gaillard [28] showed that Vina outperforms other docking software and QuickVina 2 achieves very comparable results with Vina [26]. The lower the BA score, the stronger the potential ligand is expected to bind to the receptor. It should be noted that QuickVina 2 offers only an estimation of the real BA due to its extensive use of heuristics. However, since the evolutionary design of inhibitors requires a lot of BA calculations, a balance between accuracy and computation time is important, such that one binding affinity calculation per molecule is sufficient. Although it is conceivable to perform multiple runs and average the results to achieve a more accurate estimate, this would drastically increase the computation time per molecule and result in fewer molecules being generated. Promising molecule candidates are further validated with more accurate methods (described in Section 2.4) to achieve a better estimation of their real performance.

#### 2.1.2. Synthetic Accessibility (SA)

**Figure 2:**
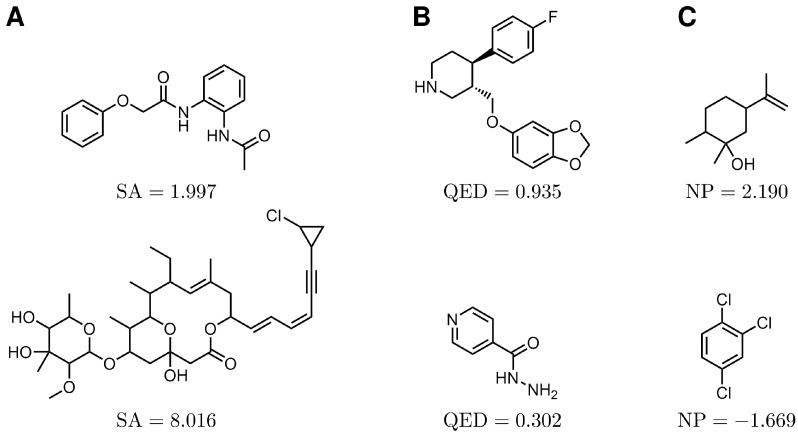
Molecules with a high and low score for each of the (**A**) SA, (**B**) QED, and (**C**) NP metrics. The top row shows molecules with an optimal score compared to molecules in the bottom row. A low SA score indicates that a molecule is easy to access synthetically [29]. A high QED or NP score indicates that a molecule has a high similarity with drug-like molecules or natural products, respectively [32].

The SA score introduced by Ertl and Schuffenhauer [29] evaluates the synthesizability of molecules by a fragment analysis of selected molecules from the PubChem Database [30,31]. A complexity score takes atypical chemical structures into account. The final SA score is the difference between the fragment score and the complexity score and ranges from 1 to 10. A lower SA score indicates easier synthetical access to a molecule. Figure 2A gives a typical example of two molecules with a high and low SA score. Synthesis is the next step in a typical drug development procedure, that follows the computer-assisted determination of the promising molecular candidates. Therefore the SA score is an important parameter to justify how complex the production of a potent drug molecule is in reality.

#### 2.1.3. Quantitative Estimate of Drug-Likeness (QED)

The QED score by Bickerton et al. [32] evaluates the drug-likeness of molecules by comparing molecular properties of a molecule and known drugs, e.g., the molecular weight, octanol-water partition coefficient, number of hydrogen bonds, and number of aromatic rings. The QED score ranges from 0 to 1, where a higher score indicates a more drug-like molecule. Figure 2B shows two molecules with a low and a high QED score. A high value of the QED metrics indicates a higher similarity to the known drug molecules and it is natural to assume that once a molecule is similar to other existing drug molecules, it is likely to posses certain properties expected in a real drug molecule.

#### 2.1.4. Natural Product-Likeness (NP)

To evaluate, if a molecule has structural characteristics like natural molecules, the NP score by Ertl et al. [33] was applied. The NP score differentiates if fragments of a molecule are natural product-like or synthetic-like. The mathematical details of the NP score are described in an earlier study [33]. The NP score ranges from −5 to 5, where a high score indicates a more natural product-like molecule. Figure 2C shows two molecules with a high and a low NP score.

#### 2.1.5. Toxicity Filter (TF)

In the proposed workflow, the drug candidates are subject to two toxicity filters: the Pan Assay Interference Compounds filter [34] and the Medical Chemical Filter described by Polykovskiy [35]. The toxicity filters evaluate if a molecule is potentially toxic due to its structural nature, e.g., the appearance of isocyanate fragments. Further, potentially unstable molecules, whose metabolites may be toxic, and charged molecules were considered. The TF score is either 0 or 1. A score of 1 indicates that the molecule passes the toxicity filters.

The framework of MOSES [35] was used to calculate the QED, NP, and SA scores and for the application of the toxicity filters. There exist other methods to obtain suitable values for the metrics than those presented here. One example is the SwissADME tool [36]. A good drug candidate is expected to have scores close to the optima for as many metrics as possible. Therefore, the EA was used to generate drug candidates with a high fitness score, which takes all five metrics into account.

### 2.2. Fitness Evaluation

The overall fitness of a potent drug molecule was calculated by using a fitness function, f(x), which was based on the molecule’s metric scores, $f_{i}$. To make the metric scores comparable, each score was scaled to be in the range from 0 (best) to 1 (worst). The BA scores were scaled with regard to the characteristic minimum value of −15 kcal/mol and maximum value of 1 kcal/mol and clipped to the range [0, 1] using the soft clipping function [37] with p=30, following

(1) $${\mathrm{SC}}_{p}\left(x\right)=\frac{1}{p}\log\left(\frac{1+e^{px}}{1+e^{p(x-1)}}\right).$$

Each molecule was assigned a single composed fitness score defined by a weighted sum:

(2) $$f\left(x\right)=\sum_{i=1}^{n}w_{i}f_{i}\left(x\right),$$

with the weights w=(0.4,0.15,0.15,0.15,0.15) and *i* corresponding to 1: BA, 2: SA, 3: QED, 4: NP, and 5: TF. The weights were chosen based on a previous study [13], where the highest attention was put on the BA metric.

### 2.3. Evolutionary Molecular Generation Algorithm

The EA used to design potential drug candidates utilizes the Simplified Molecular Input Line Entry System (SMILES) representation of the molecules in combination with a neural language model. The EA performs a randomized search in the search space of molecules, while the neural language model generates molecule fragments based on a learning process on a set of drug-like molecules. The combination of the EA and the neural language model is referred to as the evolutionary molecular generation algorithm (EMGA).

#### 2.3.1. SMILES  Representation

**Figure 3:**
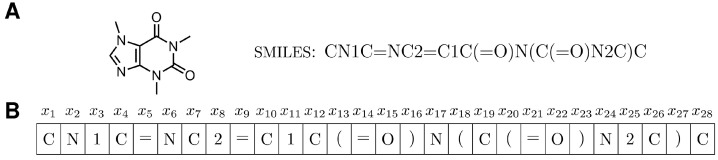
(**A**) Structural formula and SMILES string of caffeine. (**B**) Caffeine SMILES string split into a sequence of tokens $x=(x_{1},\ldots ,x_{t})$.

EMGA considers molecules in the SMILES representation. SMILES is a string-based chemical notation designed for in silico molecular research [38]. A string is a sequence of characters, which in the case of a SMILES string describes a molecule’s atoms and bonds. As an example, caffeine is shown in the SMILES representation in Figure 3A. While, in the SMILES strings, single bonds are implicit between atoms, other bonds must be specified explicitly, e.g., double bonds are represented by an equal sign, numbers describe ring structures, and brackets specify branches.

#### 2.3.2. Evolutionary Algorithm

The EA is the core algorithm of EMGA. EAs are biologically inspired population-based search heuristics. A population is a set of candidate solutions, also known as individuals. Utilizing EAs for the design of biomolecules has been demonstrated in earlier extensive studies [9,12,35,39,40,41]. The EA used in the presented study is oriented to a (μ+λ) evolution strategy [42]. After the initialization of μ random individuals, the evolutionary cycle–called generation–is repeated until a termination condition is met. In each generation, λ new offspring individuals (children) are generated by randomly choosing and mutating a parental individual; an individual is mutated by randomly deleting, adding, and replacing atoms. By passing the best performing individuals to the following generation, the quality of the molecules is expected to increase throughout evolution with respect to the fitness function.

#### 2.3.3. Neural Language Model

AI-based molecular generation models can facilitate the process of generating new and realistic drug molecules [5]. Therefore, to expectedly discover more drug-like molecules, a molecular generation model was included in EMGA. The implemented molecular generation model was based on the transformer artificial neural network. The network architecture was designed to process sequential data and contains a unique and built-in attention mechanism. The model was trained by observing a set of already known molecules, with the goal of using this set to generate molecules with similar properties.

Since the molecules in the present study are initially designed in a textual representation, i.e., as SMILES strings, the implementation of a generation model for molecular structures roots upon the concepts from the domain of language processing. A language model processes a sequence of tokens $x=(x_{1},\ldots .x_{t})$. For each token position *t*, the model is able to predict a probability distribution over the possible tokens in the sequence, conditional to the other token positions in the sequence. One example of such an approach has been given by Segler et al. [43] who demonstrated how a recurrent neural network can be used to generate molecules in their SMILES representation. In the present study, a token is the smallest building block of a SMILES string (letter, bracket, number, and equal sign) and the sequence is the SMILES string itself, see Figure 3B, i.e., the language model was trained to predict new molecules. The language model was trained iteratively by observing a set of molecules and updating the model parameters to predict the corresponding probability distributions. To enable sampling of new molecules iteratively, the generation model was trained with an autoregressive objective, i.e., the probability of the next token (letter, bracket, etc.) is conditional to the previous tokens. More formally, given a sequence of tokens describing a molecule, the likelihood function for the molecule can be factorized into conditional probabilities as

(3) $$p\left(x\right)=\prod_{i=1}^{t}p\left(x_{i}|x_{<i}\right).$$

Here, x is a sequence of tokens, *t* is the maximum number of tokes in x, and $x_{<i}$ represents all tokens in the sequence appearing before the index *i*.

**Figure 4:**
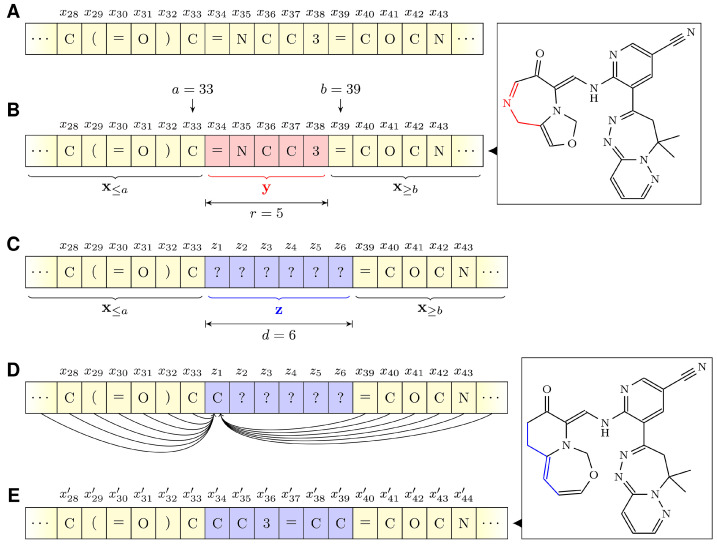
Illustration of the language model as a mutation operator. (**A**) A SMILES string to be mutated. (**B**) A random range y (red) of size *r* is selected for replacement. The top right molecular structure corresponds to the SMILES string with y highlighted in red. (**C**) The language model creates a new sequence z (blue) of length *d*. Note that d=r is not required. (**D**) Iteratively the language model calculates the $z_{i}$ values. For each $z_{i}$ all $x_{\leq a}$, $z_{<i}$, and $x_{\geq b}$ values are used as input. (**E**) After the language model processing, the resulting SMILES string is $x^{\prime }=\left(x_{\leq a}\right)z\left(x_{\geq b}\right)$. The bottom right molecular structure corresponds to the mutated SMILES string with the mutated part highlighted in blue.

The neural language model functioned as a mutation operator in EMGA, and was, therefore, able to modify existing molecules. Hence, the language model’s training objective was adjusted such that it was capable of completing contiguous parts at an arbitrary position of a SMILES string. Specifically, given a sequence of tokens x with a prefix $x_{\leq a}$ and a suffix $x_{\geq b}$ with a<b, a new sequence, $z=(z_{1},\ldots ,z_{d})$, of length *d* could be sampled such that $\left(x_{\leq a}\right)z\left(x_{\geq b}\right)$ was a valid SMILES string from the modeled distribution (see Figure 4). In contrast to training only on a left-to-right factorization order, the special transformer architecture–called XLNet [44]–was employed to maximize the likelihood of generating realistic molecules with respect to all permutations of the factorization order.

The neural language model was trained on a subset of the ZINC database [17], which contains existing and purchasable molecules. The molecules in the subset followed the definition of a drug-like molecule outlined by Polykovskiy et al. in their molecular generation benchmark paper MOSES [35]; resulting in a dataset containing 1.9 million molecules.

#### 2.3.4. Evolutionary Algorithm with Language Model

**Figure 5:**
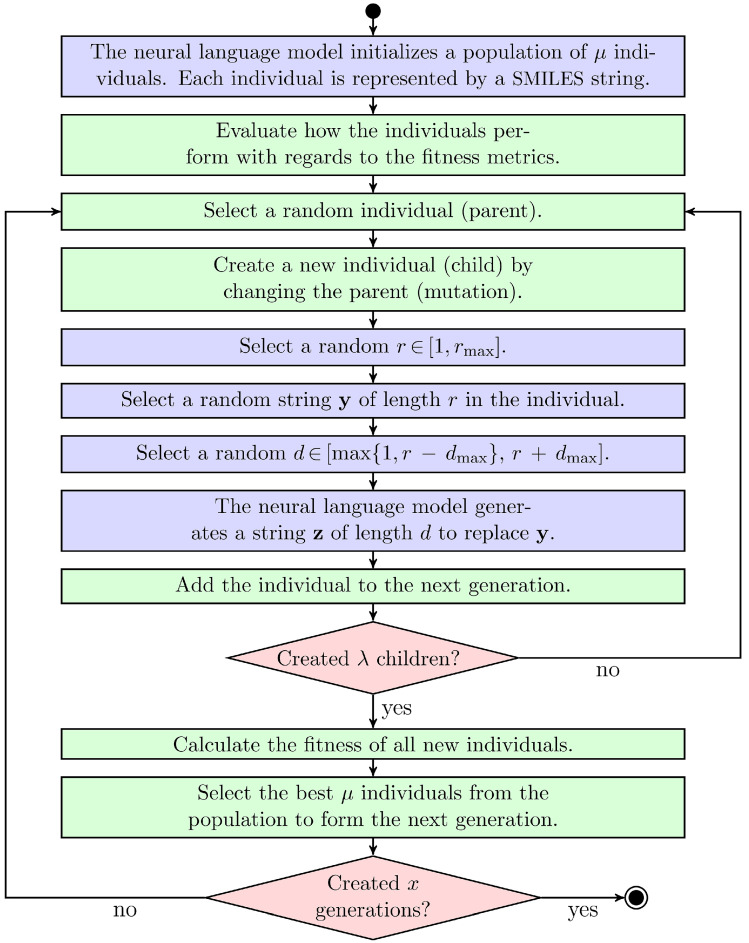
Evolutionary molecular generation algorithm (EMGA) presented in the current paper. The integrated language model and the classic EA are represented by blue and green blocks, respectively. The red boxes introduce steps where algorithmic checks are performed.

Figure 5 illustrates the workflow of EMGA. Initially, the neural language model generates a population of molecules by sampling new SMILES strings. All initial SMILES strings are sampled character by character from scratch by the language model to ensure a diverse set of starting molecules. However, starting with parts of already known structures is also conceivable to guide the evolution in a certain direction. Since the language model is trained on the ZINC database, the generated molecules should resemble the ZINC molecules and be chemically reasonable. After generation, each individual in the population is evaluated by the fitness function. Subsequently, λ individuals are created by mutating random individuals from the initial population (parents). A molecule is mutated by replacing a random part of its SMILES string with a new string using the neural language model (see Figure 4). The maximum length of the replaced string is specified by the parameter $r_{\max}$. The length of the new string may vary compared to *r*, but can maximally be $r+d_{\max}$, where $d_{\max}$ is an offset parameter. The balance between exploration of the search space and exploitation of already well-performing molecules is controlled by $r_{\max}$ and $d_{\max}$. Specifically, high $r_{\max}$ and $d_{\max}$ values can lead to diverse molecules, but also individuals being considerably different from their parents. Contrarily, small $r_{\max}$ and $d_{\max}$ values allow fine adjustments of already well-performing individuals, but also increase the risk of EMGA getting stuck in a local minimum. In the presented study, $r_{\max}$ and $d_{\max}$ were set to 8 and 5, respectively.

From the λ created individuals, the μ individuals with the best fitness scores, see Equation (2), constitute a new generation from which yet a new generation is created following the same procedure. The algorithm stops at the *x*’th generation. Here, μ and λ were set to 20 and 100, respectively, and *x* was set to 80. SMILES strings were converted into atomic coordinate files using RDKit [45] and MGLTools (https://ccsb.scripps.edu/mgltools/, accessed on 19 June 2022).

In order to illustrate EMGA at work a specific case study of M^pro^ of SARS-CoV-2 was employed. In this case, the BA score was calculated with respect to the SARS-CoV-2 M^pro^ structure (PDB ID: 6LU7 [15]) within a search space of 22Å×24Å×22Å centered around (−12Å, 15.6 Å, 69 Å), i.e., at the center of the expected drug binding site. The exhaustiveness parameter of QuickVina 2 balances the accuracy and the execution time. The exhaustiveness was kept at the default value of 8, resulting in an execution time of a few minutes per molecule.

### 2.4. Molecular Dynamics

Once the potential drug molecules were generated using EMGA, they can further be assessed through the evaluation of inhibitor binding free energy, that can be established using the Molecular Mechanics/Generalized Born Surface Area (MM/GBSA) method. In the proposed approach, the binding free energies, $G^{0}$, were calculated as

(4) $$\Delta G^{0}={\langle G_{C}\rangle }_{C}-{\langle G_{R}\rangle }_{R}-{\langle G_{L}\rangle }_{L},$$

where $G_{L}$, $G_{R}$, and $G_{C}$ are the free energies of the ligand (L), receptor (R), and the ligand–receptor complex (C), respectively. 〈.〉 indicates an average over a respective MD simulation trajectory, performed for the L, R, or C specifically [46]. These MD simulations should be carried out on the atomistic level once the suitable drug candidates are established from the EMGA calculations. The individual free energies in Equation (4) can be calculated as:

(5) $$G_{i}={\left(E_{\mathrm{MM}}+G_{p}+G_{\mathrm{np}}-TS\right)}_{i},$$

where for a selected subsystem i=L,R,C, $E_{\mathrm{MM}}$ represents the non-bonding molecular mechanics energies, $G_{p}$ and $G_{\mathrm{np}}$ are polar and non-polar solvation free energies of the *i*th subsystem, respectively, and TS accounts for the free energy associated with the entropy, *S*, of the subsystem at temperature, *T*. $G_{\mathrm{np}}$ depends on the solvent-accessible surface area, *A*, of the subsystem and a surface tension parameter $\gamma =6\times 10^{-4}\;\mathrm{kcal}/\mathrm{mol}/{Å}^{2}$ as [47,48]:

(6) $$G_{\mathrm{np}}=\gamma A.$$

The generalized Born (GB) model was used to calculate $G_{p}$ contributions in Equation (5) by employing a version of Still et al.’s [49] GB method that was modified to take into account the ionization of the solvent [50,51,52]:

(7) $$G_{p}=-k_{e}\left(\sum_{i=1}^{N}\sum_{j>i}^{N}\frac{D_{ij}q_{i}q_{j}}{g_{ij}}+\sum_{i=1}^{N}\frac{D_{ii}q_{i}^{2}}{g_{ii}}\right).$$

Here summations are performed over the *N* atoms in the corresponding subsystem (L, R, or C), $k_{e}$ is the Coulomb constant, $D_{ij}=\left(1-\frac{e^{-\kappa g_{ij}}}{\varepsilon_{s}}\right)$, $\varepsilon_{s}=74$ is the dielectric constant of the solvent, $\kappa ={\left(\frac{\varepsilon_{0}k_{B}T}{2N_{A}e^{2}I}\right)}^{-\frac{1}{2}}$ is the Debye screening length, with $k_{B}$ being the Boltzmann constant, $N_{A}$ the Avogadro number, *e* the elementary charge, I=0.15 M the ion concentration, and $\varepsilon_{0}$ the vacuum permittivity [52,53]. The function $g_{ij}$ entering Equation (7) was suggested by Still et al. [49] to have the form

(8) $$g_{ij}=\sqrt{r_{ij}^{2}+\alpha_{i}\alpha_{j}\exp\left(\frac{-r_{ij}^{2}}{4\alpha_{i}\alpha_{j}}\right)}.$$

Here, the effective Born radius, $\alpha_{i}$, indicates how deep an atom is buried inside a molecule or a protein [53,54], and can be computed following Onufriev et al. [52,53,54]. In the GB method solvent is treated as a continuum that compromises the accuracy of the molecular model compared to simulation models applying explicit solvent molecules. Furthermore, the GB method might yield varying results depending on the studied system, e.g., some GB methods underestimate $\alpha_{i}$ of atoms deeply buried inside macro-molecules [54]. However, since in the considered problem, binding free energies are calculated for the same receptor, their relative comparison is expected to be qualitatively accurate.

The entropy term in Equation (5) was computed using Schlitter’s quasi-harmonic approach [55], which provides an upper bound to the entropy as

(9) $$S≲\frac{1}{2}k_{B}\ln\det\left[I+\frac{k_{B}Te^{2}}{\hbar^{2}}M\sigma \right],$$

with *ℏ* being the reduced Planck’s constant. **M** is a 3N×3N diagonal matrix containing the atomic masses of the subsystem and σ is a covariance matrix calculated from the MD trajectory that includes the 3N coordinates describing the atoms in a given subsystem:

(10) $$\sigma_{ij}=\langle \left(\xi_{i}-\langle \xi_{i}\rangle \right)(\xi_{j}-\langle \xi_{j}\rangle )\rangle ,$$

with $\xi_{i}$ denoting the *x*-, *y*-, or *z*-coordinate of an atom. For the practical entropy calculation of the receptor, it is convenient to consider the ∼100 non-hydrogen atoms that surround the ligand, as including more atoms will make the calculation computationally too heavy.

Although the proposed AI-MD approach is general, the illustrative example of M^pro^ from SARS-CoV-2 was used for the case study to demonstrate the practical utilization of the methods. In the following, some specific details about the performed MD simulations are outlined. MD simulations were initiated based on the ligands, designed using EMGA, with the highest fitness scores. Using the Open Babel package [56], hydrogen atoms were added to the ligands, based on a pH value of 7.4, in the poses generated by QuickVina 2, and the ligand structures were minimized by the conjugate gradient algorithm with a convergence criterion of $10^{-6}$. For the simulations of the protein–ligand complex the minimized ligand structure was merged back into the receptor in the pose identified by docking. The M^pro^ was modeled using the Amber ff14SB protein force field [57] and the ligands were modeled using the general Amber force field [58]. All force fields were prepared using AmberTools [59], while simulations were carried out using NAMD2.14 [60,61] with its generalized Born implicit solvent (GBIS) functionality, which provides the solvation free energy with the electrostatic energy output. Analysis of the simulations was performed using the MDAnalysis python library [62].

Each ligand (L), receptor (R), and complex (C) simulation went through 10,000 minimization step and was afterwards simulated for 50 ns in implicit solvent. The time step was set to 1 fs, the cutoff distance of 16 Å with a switching distance of 15 Å were used for the calculation of vdW and short-range electrostatic interactions as suggested when using GBIS [52]. The temperature was kept at 310 K in all simulations by utilizing the Langevin thermostat [63] with a damping coefficient of 5 ps${}^{-1}$.

## 3. Results and Discussion

The general and versatile AI-MD algorithm for generating and selecting potent drug molecules was described above. This method was used now for an illustrative case study of M^pro^ of SARS-CoV-2. Specifically, EMGA was applied to design inhibitors of M^pro^. The evolutionary design of the inhibitors was discussed, followed by a presentation of MD simulations performed for the most promising 21 molecules, binding to M^pro^.

### 3.1. Evolutionary Design of Inhibitors

**Figure 6:**
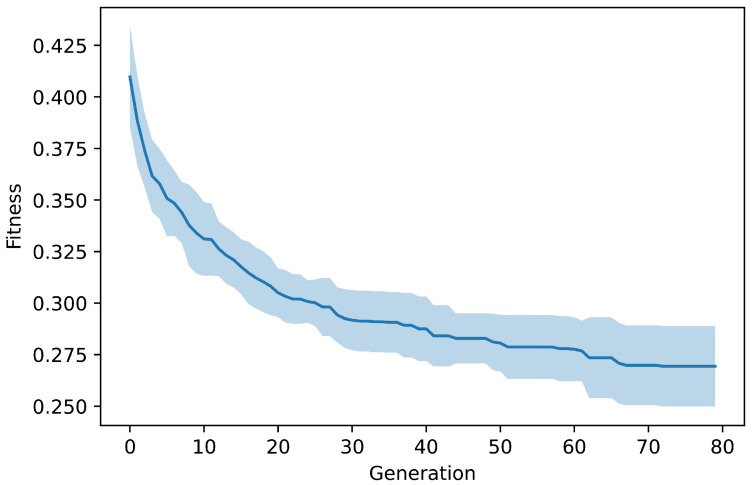
Mean values and standard deviations of the best performing individual’s fitness score in each generation calculated over 15 runs of EMGA (see Figure 5). Low fitness scores correspond to more suitable inhibitors of M^pro^.

Figure 6 shows the average fitness score of the best performing molecule from each generation based on 15 independent runs of EMGA (see Figure 5). The plot shows that EMGA optimized the starting populations towards better performing individuals. The optimization stagnated after ∼70 generations, with a fitness score of the best performing molecule being equal to 0.225. The best metric scores achieved in the last generations were for BA: −11.8 kcal/mol, QED: 0.954, NP: 0.372, and SA: 1.0. Altogether, 120,300 molecules were generated and analyzed during the 15 independent EMGA runs. In general, we found the evolutionary algorithm to be robust in regard to different configuration of μ and λ. A higher λ leads to a larger population, which can allow for a greater diversity of available molecules. However, it also increases the evaluation time of each generation thus decreases the total number of evolution steps. To increase the number of molecules for the following MD simulations, a final run of EMGA was conducted, where μ and λ were increased to 50 and 300, respectively. A list of all 144,350 generated molecules and their corresponding metric scores can be found in supplementary materials Table S1.

**Figure 7:**
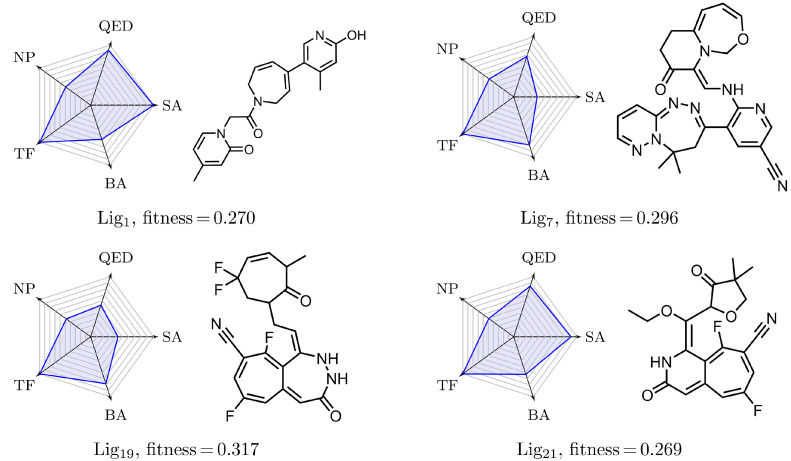
Molecular structures of four molecules, created by EMGA, with a high fitness score. Radar plots show how well the molecules perform with respect to the five metrics. The best scores are on the edge of the radar plot and the worst scores are in the center.

**Figure A1:**
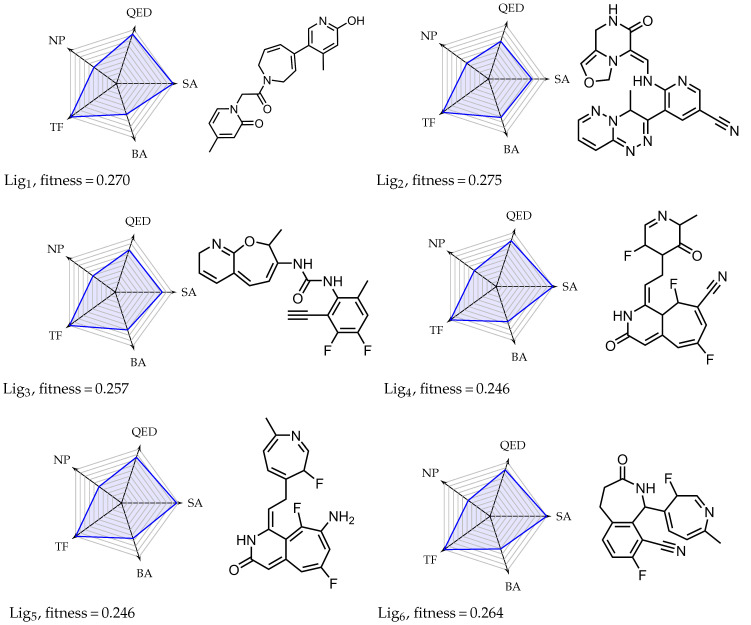

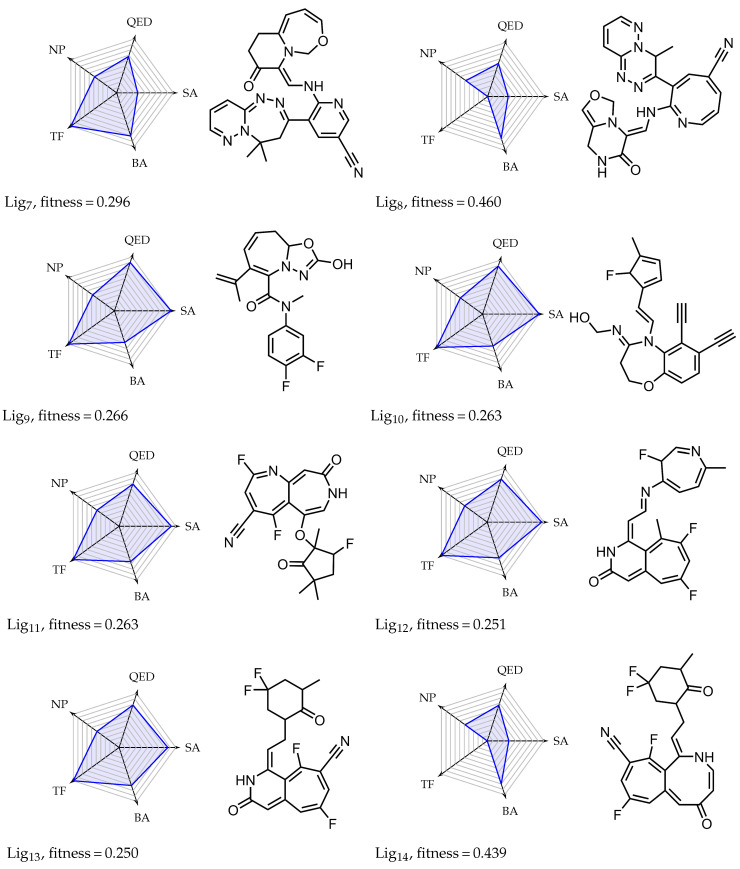

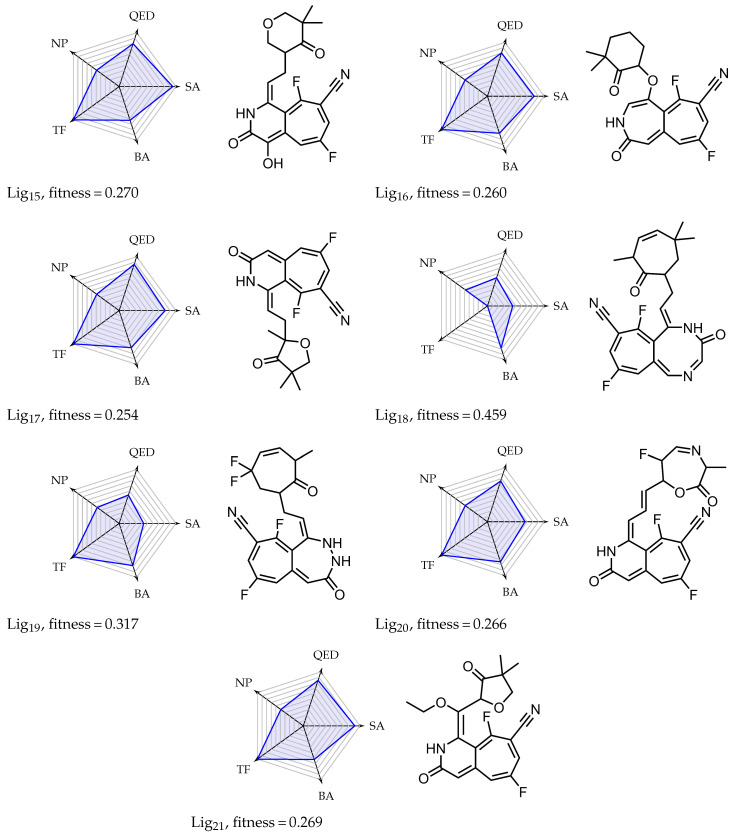
Molecular structures of simulated ligands. Radar plots show how well the ligands performed with respect to the five studied metrics. The best scores are on the edge of the radar plot and the worst scores are in the center. Molecular structures of simulated ligands. Radar plots show how well the ligands performed with respect to the five studied metrics. The best scores are on the edge of the radar plot and the worst scores are in the center. Molecular structures of simulated ligands. Radar plots show how well the ligands performed with respect to the five studied metrics. The best scores are on the edge of the radar plot and the worst scores are in the center.

From the 144,350 generated molecules, the best 200 molecules were selected based on their fitness scores and from those, 21 molecules were hand-picked based on the validity of their molecular structures. Figure 7 illustrates four molecules with high fitness scores, together with their respective radar plot. The radar plots visualize the five metric scores, with the radar edge corresponding to an optimal score. Figure A1 in Appendix A contains radar plots and molecular structures of all 21 selected molecules, and Table A1 in Appendix A lists the associated SMILES strings. Table A2 in Appendix A shows the metrics of these molecules. The best performing molecules created by the EMGA show similar structural patterns. A skeleton consisting of ring-based structures, especially nitrogen-based heterocycles such as the six-membered pyridine, pyridazine and the seven-membered azepine and diazepine rings seem to stabilize the ligand as well as favor the protease inhibition. Further, EMGA creates ligands with fluoride and cyanide as well as oxygen-based functional groups like carbonyl-, carbonamide- and hydroxyl-groups. However, carboxylate ester groups were found only rarely. These groups are known to act as electron-donors to create hydrogen bonds that would increase the BA between the ligand and the M^pro^. Similar structural patterns were also found and discussed in earlier studies [64,65].

### 3.2. Molecular Dynamics Simulations

**Figure 8:**
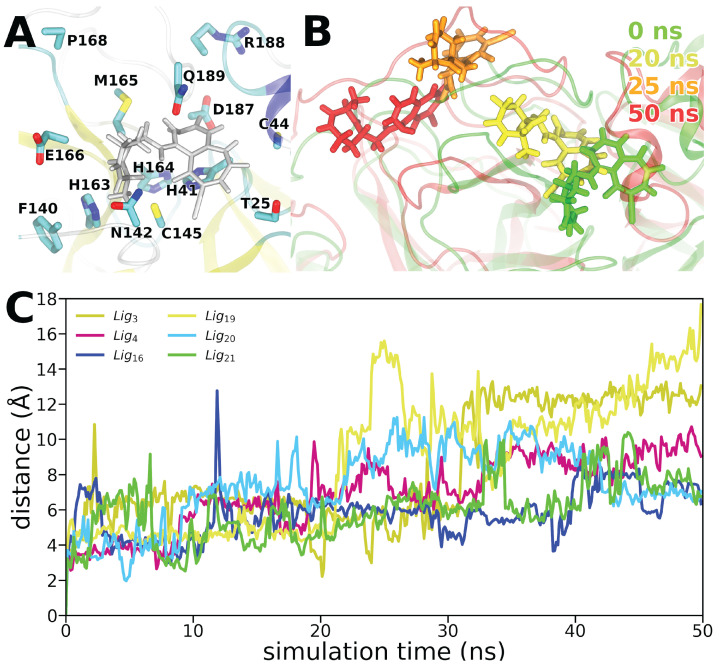
(**A**) Binding site of M^pro^ defined by the labeled residues [66] with Lig_19_ in its initial bound pose illustrated in gray. (**B**) Position of Lig_19_ in M^pro^ after 0 ns (green), 20 ns (yellow), 25 ns (orange), and 50 ns (red) of simulation. (**C**) Time evolution of the center of mass (COM) distance between the M^pro^ binding site and the ligands that drifted away from the binding site during the ligand–receptor complex simulations. Each data point was averaged over a time window of 125 ps.

**Figure A2:**
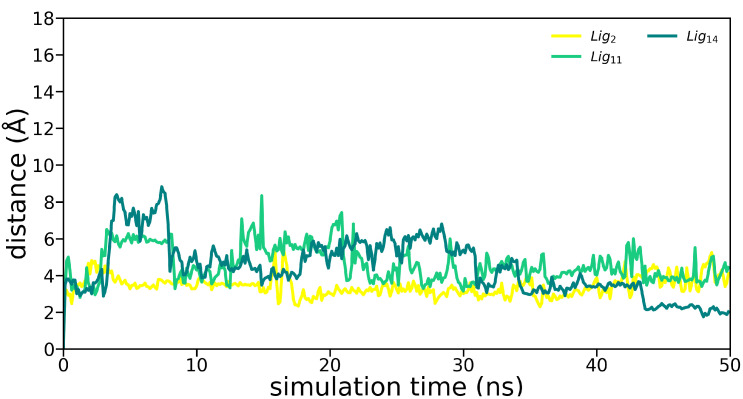
COM distance between the ligands staying closest to the binding site during the complex simulations.

**Figure A3:**
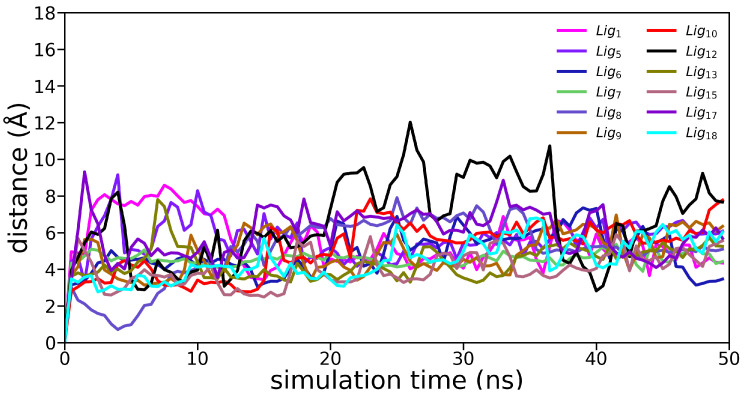
COM distance between the ligands staying within a reasonable COM distance to the binding site during the complex simulations.

To obtain binding free energies of the selected 21 ligands designed by EMGA, 43 simulations were performed. These simulations included one simulation of the empty receptor, one simulation for each ligand, and one simulation for each ligand-receptor complex. While, multiple replica simulations are advisable for more specific biophysical applications extending from the proposed methodology, the purpose here is to present the methodology. Hence, one replica of each simulation is performed. To evaluate whether the EMGA-generated ligands stayed at the M^pro^ binding site, the center of mass (COM) distance between the ligands and the binding site, defined in Figure 8A, was measured during the complex simulation. The average COM distances during the last 10 ns of the simulations are listed in Table A3 in Appendix B. Ligands Lig_3_, Lig_4_, Lig_16_, Lig_19_, Lig_20_, and Lig_21_ (see Table A1 for the SMILES nomenclature) had average COM distances above 7 Å, see Figure 8C, indicating that the respective ligands drifted away from the binding site. For illustrative purposes, one of the ligands that drifted away from the binding site, Lig_19_, is depicted at different simulation time instances in Figure 8B. Hence, ligands Lig_3_, Lig_4_, Lig_16_, Lig_19_, Lig_20_, and Lig_21_ could immediately be discarded as potential drug candidates based on the analysis of the MD simulations. Ligands Lig_2_, Lig_11_, and Lig_14_ stayed closest to the binding site with the average COM distances of 2.5–4.5 Å during the last 10 ns of the simulation, see Figure A2 in Appendix B. The MD simulations of the other studied ligands revealed their location to be around 4.5–7 Åfrom the M^pro^ binding site during the last 10 ns, see Figure A3 and Table A3 in Appendix B.

**Figure A4:**
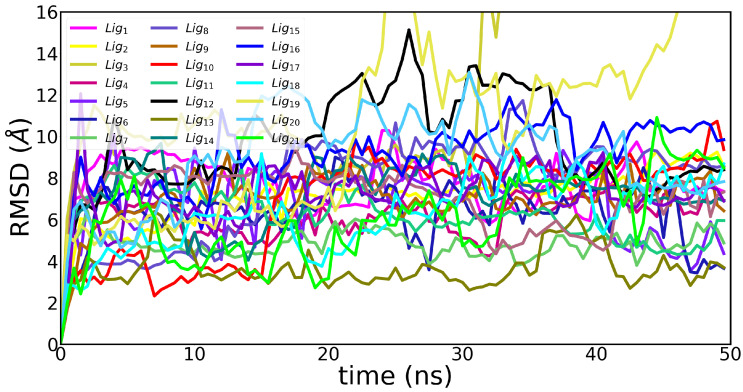
Root means square displacement (RMSD) of the simulated ligands. The RMSD is calculated based on a trajectory in which the protein backbone was aligned with itself.

**Figure A5:**
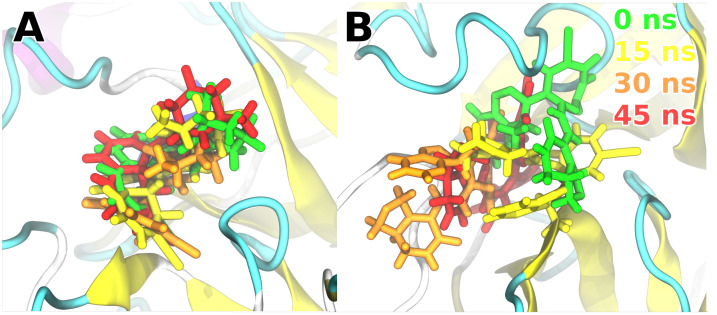
Position and orientation of ligand (**A**) Lig_13_ and (**B**) Lig_8_ in M^pro^ at simulation time instances 0 ns (green), 15 ns (yellow), 30 ns (orange), and 45 ns (red). Lig_8_ features a large RMSD value and therefore was not considered to be in a proper bound state.

Root mean square displacement (RMSD) measurements of the ligands were used to reveal information about the stability of the ligands in the binding pocket. RMSD is defined as

(11) $$\mathrm{RMSD}=\sqrt{\frac{\sum_{i=1}^{N}{\left|{\vec{r}}_{i}\left(t=0\right)-{\vec{r}}_{i}\left(t\right)\right|}^{2}}{N}},$$

where *N* is the number of atoms in a ligand and ${\vec{r}}_{i}\left(t\right)$ is the position of the *i*th atom at time instance *t*. To quantify how much the ligands move around in the binding pocket RMSD of the ligands was calculated for molecular systems, where the protein backbone was aligned with itself as it appeared at t=0 in all the MD frames. The average RMSD of the ligands during the last 30 ns of the simulations were calculated and are tabulated in Table A3 in Appendix B. Ligands Lig_1_, Lig_2_, Lig_8_, Lig_9_, Lig_10_, Lig_12_, Lig_14_, Lig_17_, and Lig_18_ had average RMSD values above 7 Å indicating that the ligand binding was not confined to a particular place in the binding pocket, see Figure A4 and Figure A5B in Appendix B. Only Lig_13_ turned out to have an RMSD value below 4 Å, suggesting that Lig_13_ is binding stably in the binding pocket, see Figure A5A in Appendix B. The ligands that move around a lot in the binding pocket cannot be considered as properly bound, and are expected to be poor drug candidates, such that the energies calculated based on Equations (4) and (5) cannot be considered as binding free energy estimates. Hence, due to their high RMSD values, ligands Lig_1_, Lig_2_, Lig_8_, Lig_9_, Lig_10_, Lig_12_, Lig_14_, Lig_17_, and Lig_18_ were discarded from the following analysis.

To obtain a measure of how much the ligands in the complex were fluctuating during the simulation time, the root means square fluctuations (RMSF) of the ligand atoms were calculated. The average RMSF of the atoms for each ligand are listed in Table A3 in Appendix B. Among the non-discarded ligands, Lig_5_, Lig_11_, and Lig_15_ had, relative to the discarded ligands, high average RMSF values in the range of 1.10–1.32 Å, while Lig_6_, Lig_7_, and Lig_13_ had low average RMSF values in the range of 0.51–0.85 Å.

**Figure A6:**
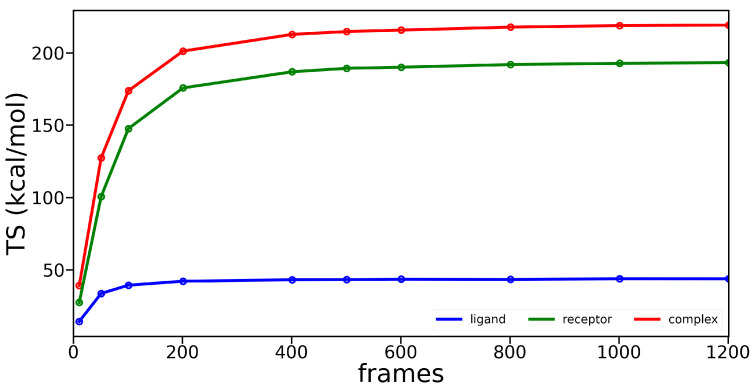
The value of the entropic contributions TS computed for Lig_9_, the receptor, and the Lig_9_-receptor complex based on the last 30 ns of the 50 ns simulation for a varying number of equally spaced simulation frames.

**Figure A7:**
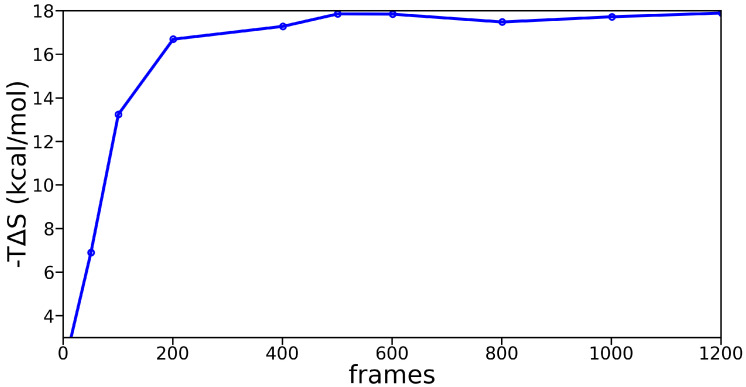
The difference in the entropic terms, TΔS, of the ligand Lig_9_, receptor, and Lig_9_-receptor complex simulations calculated based on the last 30 ns of the simulations and a varying number of equally spaced frames.

Binding free energy estimates were calculated for all the ligands that had COM distances and RMSD values below 7 Å, i.e., Lig_5_, Lig_6_, Lig_7_, Lig_11_, Lig_13_, and Lig_15_. Free energy estimates were carried out using Equations (4) and (5) based on the last 30 ns of the 50 ns simulations. Eight hundred frames were extracted from the 30 ns long MD trajectory and were used to calculate the entropy following Equation (9). According to Figure A6 and Figure A7 in the Appendix B, it is sufficient to consider 800 frames to ensure a converged entropy contribution. A resume of the binding free energy estimates is provided in Table 2. Ligands Lig_15_ and Lig_5_ have superior binding free energy estimates of −23.0 kcal/mol and −20.8 kcal/mol, respectively, which are more than twice that of the third best ligand, Lig_6_. The superior binding free energy values for the Lig_15_ and Lig_5_ ligands are mainly due to a large difference in the vdW interactions (part of $E_{\mathrm{MM}}$ in Equation (5)) between the system with the bound and unbound ligand, and being approximately −45 kcal/mol. Almost no hydrogen bonds were observed between the ligands and the receptor, highlighting that the ligand–receptor interactions predominantly are mediated through vdW interactions. Ligands Lig_7_ and Lig_11_ have positive binding free energy values implying that Lig_7_ and Lig_11_ should not spontaneously bind to M^pro^ and would likely drift away from the binding site if the simulations were extended.

Based on the MD simulations it has thus been possible to, firstly based on dynamic considerations and secondly energetic consideration, narrow down the list of ligands created by EMGA to the two promising drug candidates, namely Lig_15_ and Lig_5_. A natural next step would be to validate the potential of the identified drugs in a wet lab experiment. However, such experiments are out of the scope of the presented work.

## 4. Conclusions

A novel computational drug design workflow was introduced. The workflow applies EMGA, which is an EA combined with a neural language model-based mutation operator, and atomistic MD simulations that analyze the ligand–receptor interactions and complex stability. EMGA was designed to generate potent drug molecules, similar to those from the ZINC database and further optimize the molecules with respect to the SA, QED, NP, TF, and BA metrics. EMGA proposes drug candidates of a high expected binding affinity, thus limiting the number of necessary MD simulations that should be used to refine the list of potent drug molecules even further.

For the illustrative purpose, the proposed workflow was applied to generate drug candidates against M^pro^ of SARS-CoV-2. From the drug candidates generated by EMGA, 21 chemically valid molecules were chosen for further analysis and validation using MD simulations, which cannot only mimic the human body environment, but also yields time-resolved insight into the binding process. COM distances, RMSD values, and binding free energies between M^pro^ and the 21 ligands were computed based on the performed MD simulations. The COM distance between the ligands and the binding site and the RMSD values allowed to discard ligands based on dynamic considerations, i.e., the ligand drifting away from or moving around in the binding pocket. Binding free energy estimates provided a final ranking of the remaining ligands and showed that ligands Lig_5_ and Lig_15_ were the most promising drug candidates created by EMGA. Hence, MD simulation is an indispensable part of the proposed workflow to validate the results of EMGA. The proposed workflow has great potential, as the heuristic and data-driven proposal of realistic drug candidates complements the computationally demanding, but more accurate, MD analysis.

Although the workflow was demonstrated for the generation of inhibitors of M^pro^, it can be applied to most drug discovery problems. On the methodological level it could be interesting to adaptively configure the $r_{max}$ and $d_{max}$ parameter during the course of evolution. Higher values could provide the EA with an additional means to explore the molecular search space, while lower values could facilitate the fine-tuning of molecules that are already working well. While in general, our approach is targeted towards early stages of the drug discovery process, in the future the interesting candidates found could also be analyzed in vitro.

## Figures and Tables

**Table 1:** The range and optimal score of the binding affinity (BA), synthetic accessibility (SA), quantitative estimate of drug-likeness (QED), and natural product-likeness (NP) metrics. The toxicity filter (TF) is either 0 or 1.

	BA [kcal/mol]	SA	QED	NP	TF
score range	R	[1,10]	[0,1]	[−5,5]	{0,1}
optimum	−∞	1	1	5	1

**Table 2:** Binding free energy estimates, $\Delta G^{0}$, calculated using Equation (4) and (5). Calculation of $\Delta G^{0}$ was based on the last 30 ns of the simulations.

Ligand	$\Delta G^{0}$ (kcal/mol)
Lig_15_	−23.0
Lig_5_	−20.8
Lig_6_	−9.5
Lig_13_	−4.0
Lig_7_	5.1
Lig_11_	11.4

## Data Availability

Data are stored on institutional (university-operated) devices; they are available upon request from the corresponding authors.

## Supplementary Materials

The following supporting information can be downloaded at: https://www.mdpi.com/article/10.3390/molecules27134020/s1, Table S1, Summary of all molecules generated by EMGA together with the corresponding SMILES strings, the molecular design metrics values and the resulting fitness function.

## Abbreviations

The following abbreviations are used in this manuscript:

3CL^pro^	3C-like protease
AI	artificial intelligence
BA	binding affinity
CADD	computer-aided drug design
EA	evolutionary algorithm
EMGA	evolutionary molecular generation algorithm
LSTM	long short-term memory
MOSES	molecular sets (benchmarking plattform)
MD	molecular dynamics
M^pro^	main protease

NP	natural product-likeness
QED	quantitative estimate of drug-likeness
RMSD	root mean square displacement
SA	synthetic accessibility
SMILES	simplified molecular input line entry system
TF	toxicity filter
vdW	van der Waals

## Appendix A. Overview of Simulated Ligands

Molecular dynamics (MD) simulations were performed for 21 ligands, generated by the evolutionary molecular generation algorithm, binding to the main protease (M^pro^) of severe acute respiratory syndrome coronavirus-2. Appendix A depicts the molecular structures of the 21 ligands and radar plots representing how well each ligand performed with respect to the metrics and hence the fitness score introduced in the main paper. Table A1 lists the SMILES strings for each of the 21 ligands.

**Table A1:** Overview of the 21 ligands with their SMILES representation.

Ligand	SMILES
Lig_1_	Cc1ccn(CC(=O)N2CC=CC(c3cnc(O)cc3C)=CC2)c(=O)c1
Lig_2_	CC1C(c2cc(C#N)cnc2NC=C2C(=O)NCC3=COCN32)=NN=C2C=CC=NN21
Lig_3_	C#Cc1c(F)c(F)cc(C)c1NC(=O)NC1=CC=C2C=CCN=C2OC1C
Lig_4_	CC1N=CC(F)C(CC=c2[nH]c(=O)cc3c2=C(F)C(C#N)=CC(F)=C3)C1=O
Lig_5_	CC1=CC=C(CC=c2[nH]c(=O)cc3c2=C(F)C(N)=CC(F)=C3)C(F)C=N1
Lig_6_	CC1=CC=C(C2NC(=O)CCc3ccc(F)c(C#N)c32)C(F)C=N1
Lig_7_	CC1(C)CC(c2cc(C#N)cnc2NC=C2C(=O)CCC3=CC=COCN32)=NN=C2C=CC=NN21
Lig_8_	CC1C(C2=CC(C#N)=CC=CN=C2NC=C2C(=O)NCC3=COCN32)=NN=C2C=CC=NN21
Lig_9_	C=C(C)C1=C(C(=O)N(C)c2ccc(F)c(F)c2)N2N=C(O)OC2CC=C1
Lig_10_	CC1=CC=C(C=CN2C(=NCO)CCOc3ccc(C#N)c(C#N)c32)C1F
Lig_11_	CC1(C)CC(F)C(C)(OC2=CNC(=O)C=C3N=C(F)C=C(C#N)C(F)=C32)C1=O
Lig_12_	CC1=CC=C(N=CC=c2[nH]c(=O)cc3c2=C(C)C(F)=CC(F)=C3)C(F)C=N1
Lig_13_	CC1CC(F)(F)CC(CC=c2[nH]c(=O)cc3c2=C(F)C(C#N)=CC(F)=C3)C1=O
Lig_14_	CC1CC(F)(F)CC(CC=C2NC=CC(=O)C=C3C=C(F)C=C(C#N)C(F)=C32)C1=O
Lig_15_	CC1(C)COCC(CC=c2[nH]c(=O)c(O)c3c2=C(F)C(C#N)=CC(F)=C3)C1=O
Lig_16_	CC1(C)CCCC(OC2=CNC(=O)C=C3C=C(F)C=C(C#N)C(F)=C32)C1=O
Lig_17_	CC1(C)COC(C)(CC=c2[nH]c(=O)cc3c2=C(F)C(C#N)=CC(F)=C3)C1=O
Lig_18_	CC1C=CC(F)(F)CC(CC=C2NC(=O)C=NC=C3C=C(F)C=C(C#N)C(F)=C32)C1=O
Lig_19_	CC1C=CC(F)(F)CC(CC=C2NNC(=O)C=C3C=C(F)C=C(C#N)C(F)=C32)C1=O
Lig_20_	CC1N=CC(F)C(C=CC=c2[nH]c(=O)cc3c2=C(F)C(C#N)=CC(F)=C3)OC1=O
Lig_21_	CCOC(=c1[nH]c(=O)cc2c1=C(F)C(C#N)=CC(F)=C2)C1OCC(C)(C)C1=O

**Table A2:** The molecular metrics of the 21 ligands. These metrics are described in Section 2.1.

Ligand	BA	SA	QED	NP	TF
Lig_1_	−8.1	1.019	0.918	−0.089	1
Lig_2_	−10.4	3.119	0.699	−0.272	1
Lig_3_	−10.2	2.543	0.783	−0.133	1
Lig_4_	−9.4	1.000	0.850	−0.212	1
Lig_5_	−9.5	1.254	0.848	−0.094	1
Lig_6_	−8.7	1.000	0.861	−0.256	1
Lig_7_	−11.8	6.664	0.676	−0.174	1
Lig_8_	−11.4	6.745	0.615	−0.238	0
Lig_9_	−8.4	1.000	0.898	−0.248	1
Lig_10_	−8.5	1.000	0.890	−0.149	1
Lig_11_	−9.5	1.695	0.783	−0.183	1
Lig_12_	−9.6	1.342	0.799	−0.082	1
Lig_13_	−10.3	2.252	0.787	−0.183	1
Lig_14_	−11.8	6.567	0.662	−0.174	0
Lig_15_	−9.0	1.477	0.796	−0.083	1
Lig_16_	−10.0	2.586	0.803	−0.118	1
Lig_17_	−10.0	2.692	0.858	−0.097	1
Lig_18_	−11.5	5.985	0.523	−0.235	0
Lig_19_	−11.5	6.102	0.527	−0.209	1
Lig_20_	−10.9	4.036	0.756	−0.149	1
Lig_21_	−9.0	1.813	0.844	−0.103	1

## Appendix B. Molecular Dynamics Analysis

The time evolution of the distances between the center of mass (COM) of selected ligands and the COM of the binding site during the complex simulations is depicted in Figure A2 and Figure A3. Figure A4 shows the time evolution of the RMSD values for the ligands, while Figure A5 shows the placement of the Lig_8_ and Lig_13_ ligands inside M^pro^, where Lig_8_ and Lig_13_ have a high and a low average RMSD value, respectively, at different simulation instances. Figure A6 shows how the ligand, receptor, and complex entropic term TS depends on the number of simulation frames included in the entropy calculation, see Equation (9) in the main paper. Figure A7 shows the difference in TS between the complex and the receptor and ligand. Figure A6 and Figure A7 demonstrate that the entropy converges when more than 500 frames are used in the calculation.

**Table A3:** Average COM distances between the ligands and the binding site are measured during the last 10 ns simulation of the ligand–receptor (complex) simulations. Average RMSD values of the ligands are calculated based the last 30 ns the complex simulations with the protein backbone aligned with itself. RMSF values are calculated as an average over the RMSF of each atom in the ligands throughout the 50 ns simulations of the complex. Ligands discarded due to high COM and RMSD values are highlighted in orange and yellow, respectively.

Ligand	avg. COM (Å)	avg. RMSD (Å)	avg. RMSF (Å)
Lig_1_	4.82	7.66	1.37
Lig_2_	3.83	7.80	1.05
Lig_3_	12.43	14.05	0.99
Lig_4_	9.08	6.40	1.03
Lig_5_	6.15	6.90	1.15
Lig_6_	4.70	6.46	0.64
Lig_7_	4.75	4.85	0.51
Lig_8_	5.00	9.13	1.07
Lig_9_	5.87	7.17	0.39
Lig_10_	6.20	8.59	0.63
Lig_11_	4.23	5.76	1.32
Lig_12_	6.82	10.44	1.34
Lig_13_	5.04	3.68	0.85
Lig_14_	2.62	7.67	1.18
Lig_15_	4.85	6.57	1.10
Lig_16_	7.33	9.95	0.83
Lig_17_	5.24	7.56	0.45
Lig_18_	5.55	7.19	1.11
Lig_19_	13.28	13.42	1.15
Lig_20_	7.27	9.50	1.54
Lig_21_	7.93	7.15	0.71
